# Entropy production with the flow of nanomaterials through the permeable stretched surface with heterogeneous–homogenous chemical reaction

**DOI:** 10.1039/d3na00639e

**Published:** 2023-09-26

**Authors:** Hashim, Sohail Rehman, Serhan Alshammari, Ahmed Osman Ibrahim, Naeem Ullah

**Affiliations:** a Department of Mathematics & Statistics, The University of Haripur Haripur 22620 Pakistan; b Department of Mathematics, Islamia College Peshawar 25120 Khyber Pakhtunkhawa Pakistan sohail08ktk@gmail.com; c Industrial Engineering Department, College of Engineering, University of Ha'il Ha'il 55476 Kingdom of Saudi Arabia; d Department of Architectural Engineering, College of Engineering, University of Ha'il Ha'il 55476 Kingdom of Saudi Arabia

## Abstract

In various thermodynamic procedures and the optimisation of thermal manipulation, nanofluids flowing through porous media represent an emerging perspective. The main objective of this study, from the perspective of thermal applications, was the investigation of the flow of nanofluid over a horizontal stretched surface embedded in a porous medium. The effects of the chemical reactions on the surface, magnetic field, and thermal radiations were invoked in the mathematical formulation. The non-Darcy model examines the fluid flow in the porous media. The principles of thermodynamics were employed to integrate entropy optimisation methods with the established theoretical approach to analyse the thermal behaviour of nanomaterials in the chemical reactive diffusion processes. Using the Tiwari-Das nanofluid model, the volume fraction of the nanomaterials was merged in the mathematical equation for the flow model. Water was taken as a base fluid and nanoparticles composed of aluminium oxide (Al_2_O_3_) and silver (Ag) were used. The significance of radiation, heat production, and ohmic heating were included in the energy equation. Furthermore, an innovative mathematical model for the diffusion of the autocatalytic reactive species in the boundary layer flow was developed for a linear horizontally stretched surface embedded in a homogeneous non-Darcy porous medium saturated with the nanofluid. The computer-based built-in bvp5c method was used to compute numerically these equations for varied parameters. It is clear that the magnetic parameter has a reversal influence on the entropy rate and velocity. Temperature and velocity are affected in the opposite direction from a higher volume fraction estimate. Thermal field and entropy were increased when the radiation action intensified. The inclusion of nanoparticle fraction by the volume fraction of nanoparticles and Brinkman number also enhances the system entropy. Entropy production can be minimized with the involvement of the porosity factor within the surface.

## Abstract 2

In various thermodynamic procedures and the optimisation of thermal manipulation, nanofluids flowing through porous media represent an emerging perspective.

## Graphical abstract

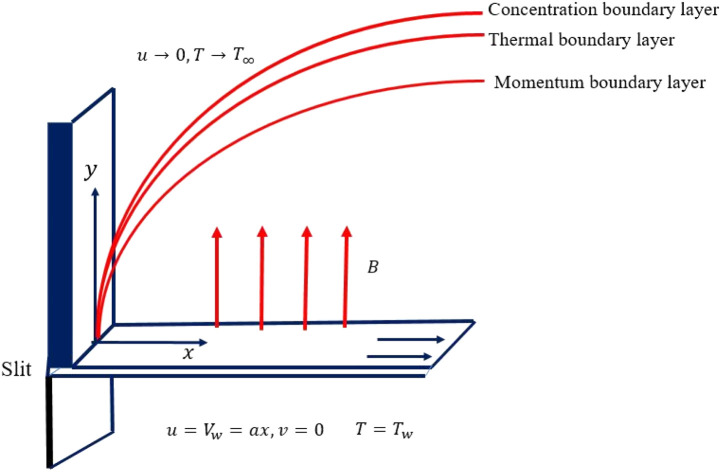

## 1. Introduction

Under diverse physical conditions, thermal energy is transported primarily through conduction, convection, and radiation. Radiation has a number of advantages over the other two modes of heat transfer, conduction and convection.^1^ Electromagnetic waves are capable of moving through a vacuum because of the radiative heat flux. For heat transport, both conduction and convection require a medium. Convective or conductive heat transport depends linearly on a temperature difference, while radiant heat transport is proportional to the difference in the fourth power of temperature. Radiative heat transfer is increasingly essential at higher temperatures and is used in a variety of engineering applications, including combustion,^2^ nuclear power technology,^3^ atmospheric reentry,^4^ and solar energy.^5^ Convection and conduction are manifestations of short-range phenomena that are governed by the conservation of energy across any small volume. In contrast to the long-range phenomenon of radiation, the emission, absorption, and reflection from the wall will immediately alter the total radiative heat transfer. Every object with a temperature greater than 0 K naturally emits thermal radiation, which has been extensively employed in energy harvesting, thermal transfer, and management.^6^ A specific method of transferring energy between two objects *via* thermal radiation is known as radiative heat transfer. Radiant heat (RH) is referred to as far-field radiative heat transfer when the distance between the heat source and the heat sink is significantly greater than the characteristic thermal wavelength.^7^ The Stefan–Boltzmann law limits the radiative heat flux, which is primarily determined by propagating waves. Coupled models have significant problems with radiative heat transfer for combustion applications. This heat transport mechanism is the most common and dictates accurate modelling in environments with high temperatures and concentrations of radiative species.

The heat energy generated while using various equipment and devices in both small and large sizes must be released into the environment in order to prevent overheating and improve efficiency. Most thermal exchangers use fluids to remove the heat generated while a machine is operational. The use of common fluids in the process of heat exchange is less acceptable due to their poor thermal conductivity. Recently, researchers created a groundbreaking method to enhance the thermal characteristics and thermal energy exchange rate of common fluids. Due to its importance in a variety of scientific fields and the industrial sector, nanotechnology has recently become one of the most significant research areas. Researchers are closely examining the varied thermal characteristics of such nano-sized particles due to the importance of nanotechnology in thermal design and multifaceted sciences. In order to meet the rising demand for energy resources, resulting from technological appliances, modern nanotechnology innovations combine nanoparticle communications to optimize the heat/mass transfer mechanism. They mixed ordinary fluid with metal oxides and nanoparticles of several metals. Typically, nanoparticles are divided into two phases: a liquid phase made up of the base fluid and a solid phase made up of the nanomaterials. Nanofluids are more sophisticated than ordinary fluids since they have unique properties and precise specifications. A variety of unique characteristics of nanofluids include increased viscosity, thermal conductivity, and transfers of heat. Many metallic and non-metallic nanofluid models have been studied because of their improved thermal performance. The first pioneering study to introduce nanofluids was performed by Choi.^8^ Afterward, Eastman *et al.*^9^ studied the thermal conductivity and heat transmission of nanofluids. Shafiq *et al.*^10^ studied the bioconvective tangent hyperbolic magnetohydrodynamics (MHD) nanofluid flow with Newtonian heating. In order to study the effects of chemical radiation on a spherical-shaped cylinder encased in a porous medium, Tlili *et al.*^11^ used multiple slip effects on an MHD non-Newtonian nanofluid. Haider *et al.*^12^ theoretically and numerically explored the impact of the nanoparticle shape on a water-based aluminium oxide nanofluid moving towards a solid cylinder with the consequences of heat transfer. Khamliche *et al.*^13^ used Cu nanoparticles to create high thermal conductivity nanofluids. Gangadhar *et al.*^14^ examined the two-dimensional boundary flow layer of a nanofluid over a stretched surface using the spectrum relaxation method. They concluded that when Prandtl numbers increase, the temperature and the nanoparticle volumetric fraction decrease. Furthermore, they concluded that with increased values of the heat absorption parameter, the local Nusselt number increases. Furthermore, Kotha *et al.*^15^ and Gangadhar *et al.*^16^ reported that gyrotactic microorganisms are contained on the boundary layer surface by a nanofluid flow. In-depth study on the properties of nanofluids can be found in the literature.^17–20^

Entropy generation is a well-known indicator of the degree of irreversibilities present throughout any heating process. Cooling and heating are significant events in many engineering procedures and industrial sectors, particularly in the fields of energy and electronics. Entropy coproduction must be optimized to avoid irreversible losses that could impair system performance. Bejan^21^ initially established the Bejan number (Be) as the ratio of heat irreversibility to the total loss of heat due to fluid frictional forces in order to figure out the generation of entropy. The second rule of thermodynamics to estimate the system irreversibilities allowed us to access the most practicable design of thermal systems.^22,23^ The presence of irreversibilities reduces the performance of an engineering device, and the level of the availability of this factor in a process is gauged by the entropy generation function. For the energy systems to be designed with the best possible efficiency, the entropy formation must be reduced because it serves as the measurement criterion for the available work destruction of the systems.^24^ Moreover, the formation of entropy results in a reduction in the useable power cycle outputs of a power production device, and an increase in the power input requires the cycle to produce power. It is crucial to highlight that the investigations based on the second rule of thermodynamics are more trustworthy than those analyses based on the first law due to the first law's efficiency limitations in heat transfer engineering systems.^25^ The analysis of entropy establishment is carried out to boost system performance. Moreover, entropy formation can occur by mass transfer, heat transfer, viscous dissipation, and finite temperature gradients.^26,27^

With the broad spectrum of applications in chemical engineering, the study of heat transport in the stretched flow is quite significant. In the current study, we considered the convective heat transfer coating flow through the boundary layer of a magnetic nanofluid over an expanding slit dipped in a porous medium under the magnetic field. The inquiry is unique in the sense that it involves multiple phenomena, including radiative flux, energy source, porosity characteristics, and dual chemical reactions. The flow pattern is curvilinear when the medium porosity is approximately one, and the inertial effect is brought on by the curvature of the path. The streamlined bend and drag rise quickly with velocity. A Tiwari-Das mechanism, which accounts for the volume fraction and is less complex than the KKL nanoscale model,^28^ was used to study the nanoparticles and analyze their nanoscale behavior. Additionally, the Tiwari-Das model enables us to examine the nanoparticle material types, in contrast to the Buongiorno model.^29^ Both nanoparticles are of homogenous size and shape. The analysis of entropy production for the MHD flow in the presence of a non-Darcy-Forchheimer medium with radiative heat flux and dual chemical reaction on the sheet surface has not yet been discussed. Therefore, the consideration of irreversibility in the flow of radiative heat flux, non-Darcy porous medium, and Lorentz force towards a porous surface is the novelty of this research. Two different nanoparticles silver Ag and aluminum oxide Al_2_O_3_ were suspended in water. The current study incorporates viscous heating and ohmic dissipation to create realistic temperature distribution estimations for chemical processes. The conservation relations for mass, energy, momentum, and diffusion (for species *X̂* and *Ŷ*) are converted into a system of non-linear coupled higher-ordered differential equations using the proper scaling variables. The MATLAB bvp5c procedure uses four-point Gauss–Lobotto formulas to solve the developing nonlinear ordinary differential boundary value problem.^30^ The dimensionless quantities exhibit an extensive display of velocity, temperature, species *X̂* and *Ŷ*) local Sherwood numbers, frictional coefficient, heat transport coefficient, and concentrations of *X̂* and *Ŷ* species. The study is pertinent to the industrial fluid dynamics of stretching surface engineering components with smart magnetic nano-coating at high temperatures. According to our understanding, the current study is the first to address smart nanofluid dynamics.

## 2. Quantification and thermophysical features of nanoparticles

In this physical problem, water serves as the base fluid and includes the mixing of Ag and Al_2_O_3_ nanoparticles. In this investigation, the volume fraction of Ag was set at 1%, and the solid volume fraction of Al_2_O_3_ ranged from 1% to 5%, *i.e.*, *φ* = *φ*_Ag_ + *φ*_Al_2_O_3__. The material property ratios are defined here by following the resultant representations:1
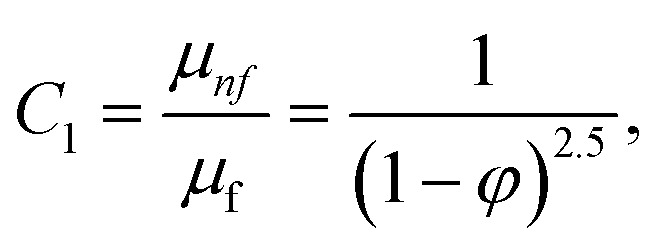
2
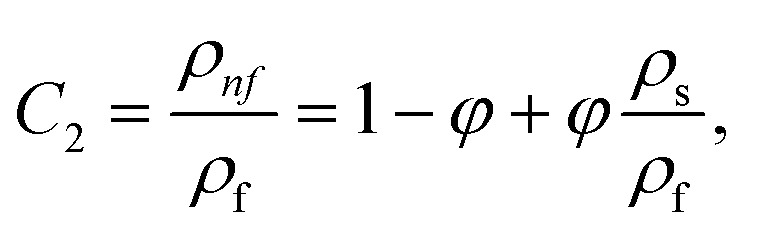
3
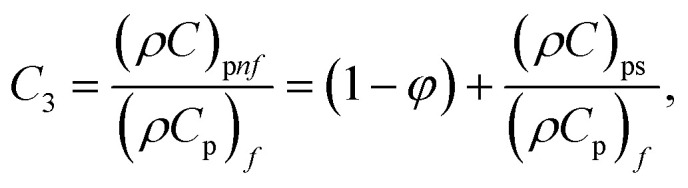
4
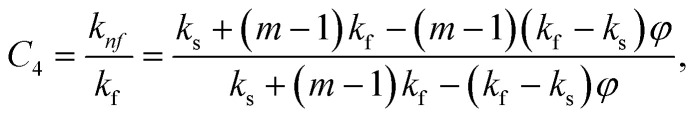
5
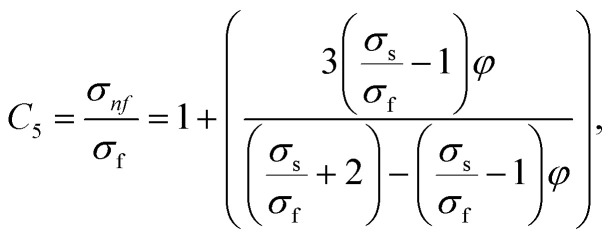


The volumetric heat capacities of solid nanoparticles and base fluids are indicated in the aforementioned expression by (*ρC*_p_)_*nf*_ and (*ρC*_p_)_*f*_. The subscripts *s*, *f*, and *p* stand for solid particles, base fluid, and nanoparticles, respectively. *φ* stands for the volumetric percentage of nanoparticles. *m* stands for the form factor of nanoparticles.

This inquiry takes into account the solid nanoparticles of silver (Ag) and aluminum oxide (Al_2_O_3_), while water is taken as a base fluid. The numerical values of the nanoparticles are shown in Table 1.

**Table 1:** Thermophysical properties of the nanomaterials

Physical characteristic	Base fluid (water)	Al_2_O_3_	Ag
*ρ* (kg m^−3^)	1050	3970	10 500
*c*_p_ (J kg^−1^ K^−1^)	3617	765	235
*k* (Wm^−1^ K^−1^)	0.492	40	429
*σ* (S m)^−1^	0.05	35 × 10^5^	6.30 × 10^7^
Pr	6.2	—	—

## 3. Modelling and mathematical formulation

**Fig. 1:**
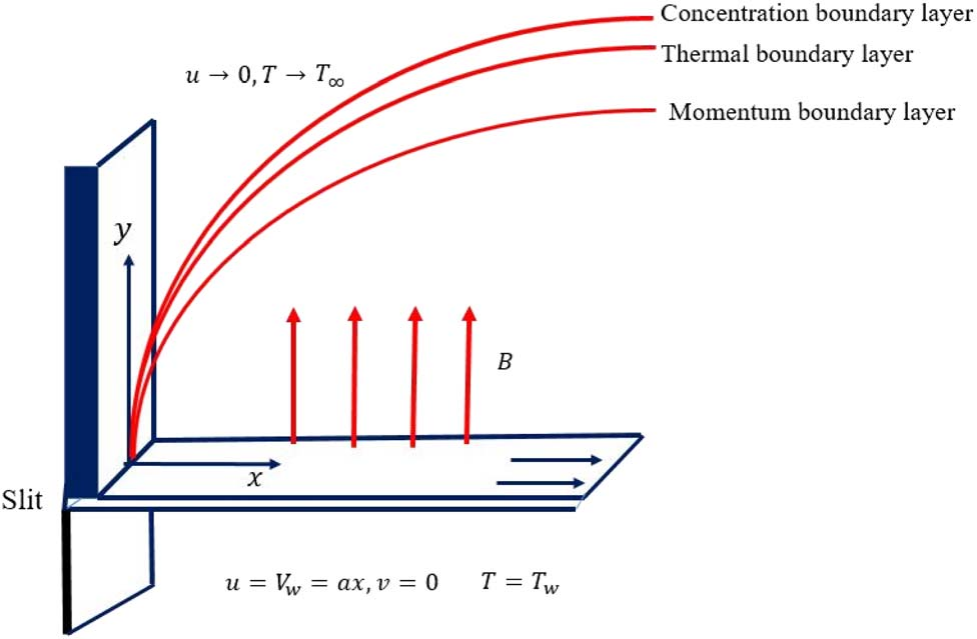
Diagrammatic interpretation of the flow problem.

We considered the motion of a viscous nanofluid through a stretched surface corresponding to the plane *y* = 0 with steady and laminar fluid layers. As can be seen in Fig. 1, the flow is restricted to the region *y* > 0, where *y* is the coordinate measured perpendicular to the stretched surface. The first-order homogeneous and heterogeneous chemical species reaction processes are included in this study. The particles, *i.e.*, silver (Ag) and aluminum oxide (Al_2_O_3_), are considered to be nanomaterials. The plate was stretched along the *x*-axis while experiencing the linear velocity *u* = *V*_w_ = *ax*, where *a* is a positive constant. Along the *y*-axis, a magnetic field *B* was delivered at a constant strength (*i.e.*, normal to the flow direction). According to general belief, the magnetic Reynolds number is low. As a result, in comparison to the magnetic field that is provided externally, the induced magnetic field is rather moderate.

Herein, we use the straightforward boundary layer flow model proposed by Chaudhary and Merkin^31,32^ for the interface of homogenous–heterogeneous processes involving the dual chemical species *X̂* and *Ŷ*:

6 *X̂* + 2*Ŷ* → 3*Ŷ*, rate *K*_1_*cd*^2^,

7 *X̂* → *Ŷ*, rate *K*_s_*c*.

Herein, *K*_1_ and *K*_s_ are the corresponding homogeneous and heterogeneous reaction rate constants, while *d* and *c* are the concentrations of the chemical species *X̂* and *Ŷ*. The thickness of the nanoliquid boundary layers is considered to be high. Radiative heat transport is approximated using the Rosseland diffusion algebraic approximation. Radiation is emitted at the stretched surface. Viscous heating due to fluid molecule and nanoparticle interaction is added to the formulation. The resulting conservation equations using a Cartesian (*x*, *y*) coordinate system, with the involvement of ohmic dissipation (Joule heating) affects porosity factor and heat source, are as follows:^33–35^8
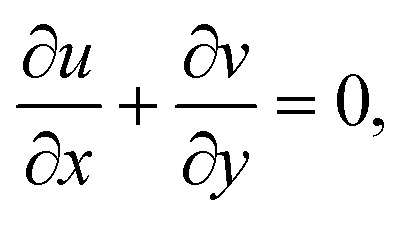
9
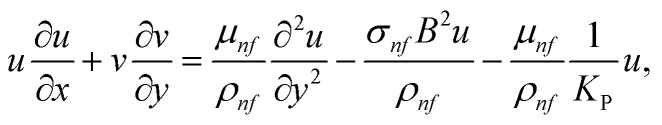
10
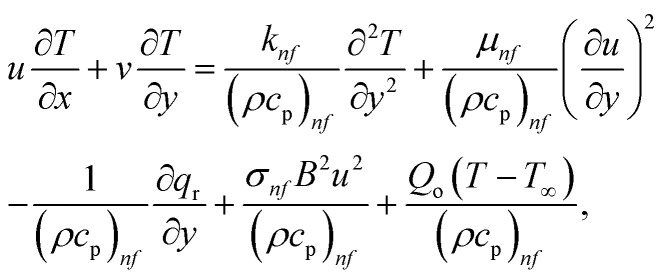


Since the reaction is irreversible, the reaction rate declines for external flow as well as for the boundary layer. Thus, the mathematical expression for species *X̂* and *Ŷ* occurs as.^36,37^11
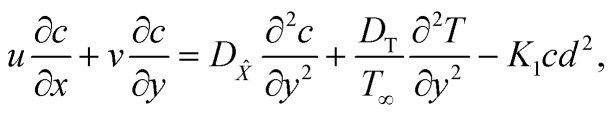
12
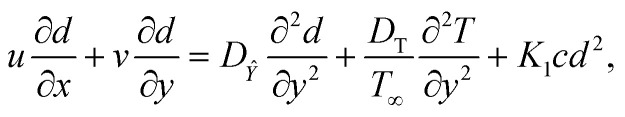


with the following boundary conditions13
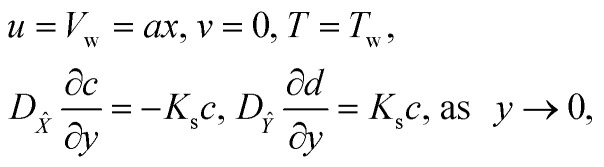

14 *u* = 0, *T* = *T*_∞_, *c* → *c*_∞_, *d* → 0, as *y* → ∞,

Using similarity transformations15
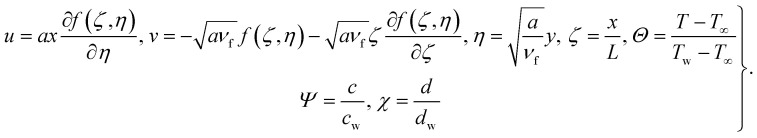


We have16
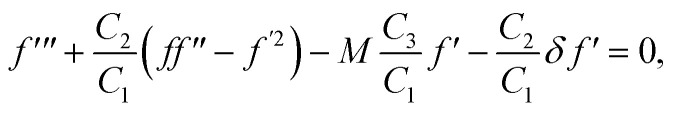
17
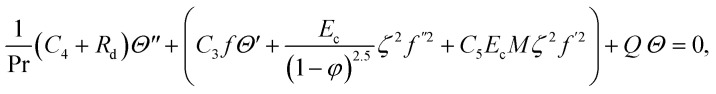

18 *Ψ*′′ + Sc*Ψ*′*f* − Sc*KΨχ*^2^ = 0,

19 *εχ*′′ + Sc*χ*′*f* + Sc*KΨχ*^2^ = 0,

20 *f*′(*ζ*,0) = 1, *f*(*ζ*,0) = 0, *Θ*(*ζ*,0) = 1, *Ψ*′(*ζ*,0) = *K*_C_*Ψ*, *εχ*′(*ζ*,0) = −*K*_C_*Ψ*,

21 *f*′(*ζ*,∞) = 0, *Θ*(*ζ*,∞) = 0, *Ψ*(*ζ*,∞) = 1, *χ*(*ζ*,∞) = 0,

We anticipate that the diffusion coefficients of the chemical species *X̂* and *Ŷ* are approximately equivalent in size in the majority of applications. Therefore, the ratio of diffusion coefficients was supposed to be one (*ε* = 1), leading to the assumption that the diffusion coefficients *D*_*X̂*_ and *D*_*Ŷ*_ are of identical size, *i.e.*, *D*_*X̂*_ = *D*_*Ŷ*_. Following this relation, we have

22 *Ψ* + *χ* = 1, or *Ψ* = 1 − *χ*.

Eqn (18) and (19) gives

23 *Ψ*′′ + Sc*Ψ*′*f* − Sc*KΨ*(1 − *Ψ*)^2^ = 0,

24 *Ψ*′(*ζ*,0) = *K*_C_*Ψ*(*ζ*,0), *Ψ*(*ζ*,∞) = 1.

### 3.1. Physical quantities

Skin friction and Nusselt number25
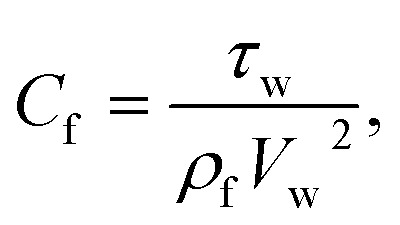
26
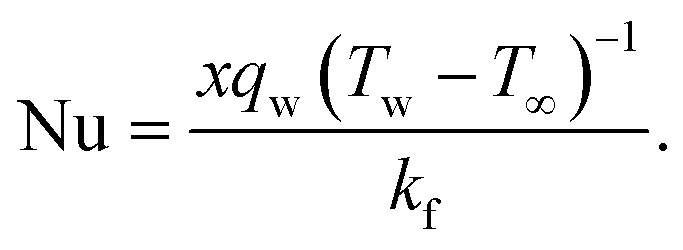


Where the momentum and energy flux at the interface are defined as:27
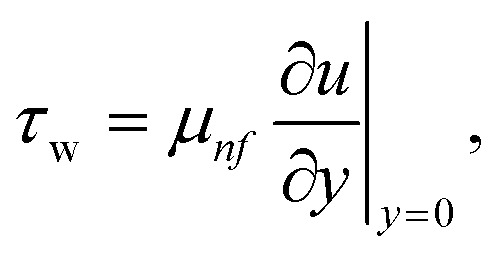
28
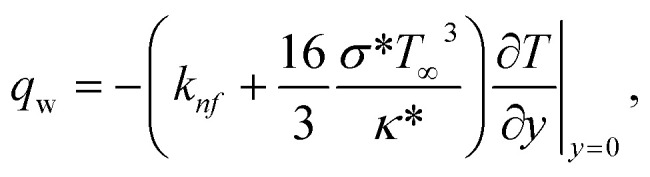


Using eqn (15), the dimensionless form of physical quantities are:29
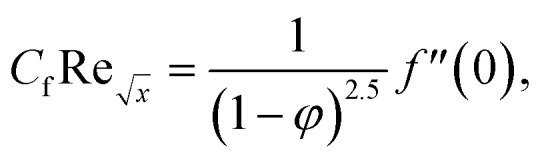
30
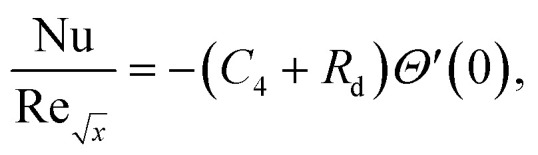


The apparent mass flux for species *X̂* and *Ŷ* is demarcated as:^38^31
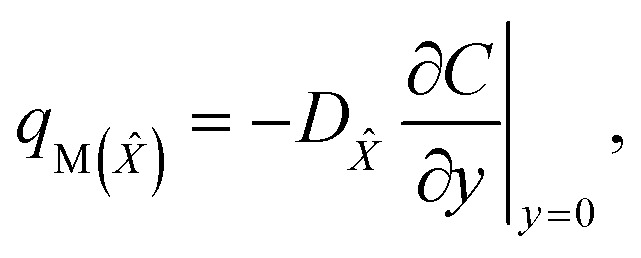
32
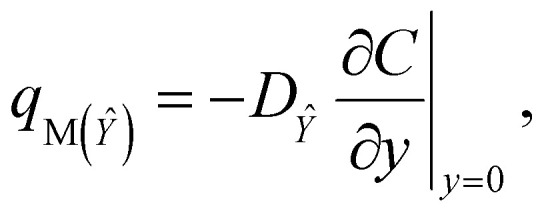


The Sherwood numbers define the mass fluxes for mass species *X̂* and *Ŷ*, *i.e.*, the normalized mass transfer rates at the stretched sheet surface, as follows:33
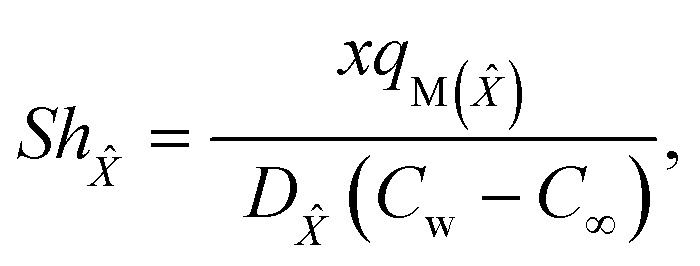
34
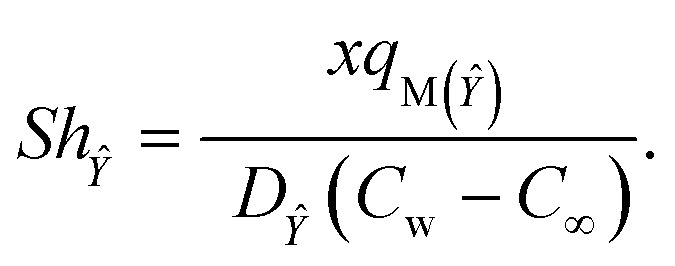


The dimensionless forms of eqn (33) and (34) are

35 *Sh*_*X̂*_ = −*Ψ*′(0),

36 *Sh*_*Ŷ*_ = −*χ*′(0),

The present study generalizes analyses from previous studies because they did not take into account Sherwood number behavior in their research.

### 3.2. Entropy modelling

The irreversible process leads to entropy generation optimization. In the entropy production analysis, the viscous dissipation, Joule heating, radiant heat flux, and heterogeneous–homogeneous chemical reaction effects are incorporated. One of the primary goals of the entropy generation is to gauge the irreversibility of any system. The five factors that cause entropy creation are the temperature differential, magnetic field, porosity of the medium, viscous dissipation, and species diffusion. Under these circumstances, the dimensional form entropy is provided in ref. 39–41.37
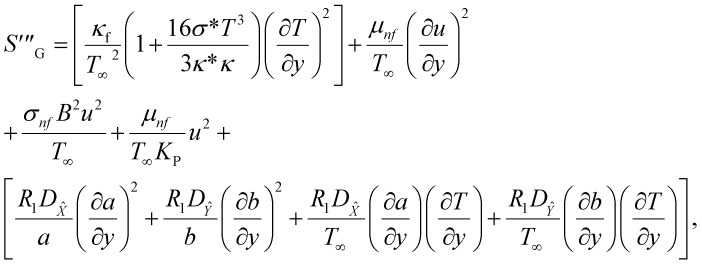
Here, the first to fifth terms on the right hand side of eqn (37) are the entropy generation due to heat transfer, viscous dissipation, Joule heating (magnetic field), porosity factor, and concentration of the *X̂* and *Ŷ* species, respectively.

Furthermore,38
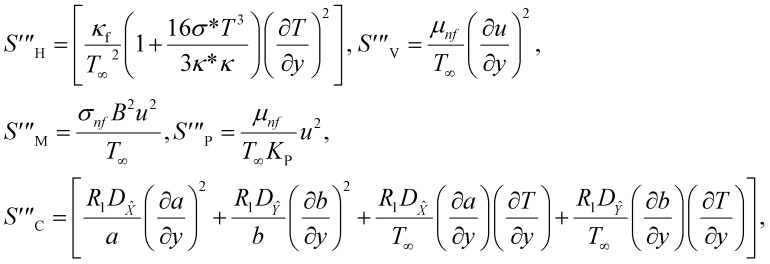
39
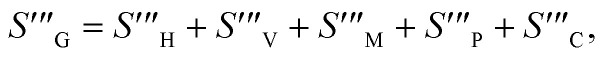


The overall entropy, abbreviated in the text for global thermodynamics, was obtained by integrating these local values over the entire volume. The following equations can be used to express them:^42^40
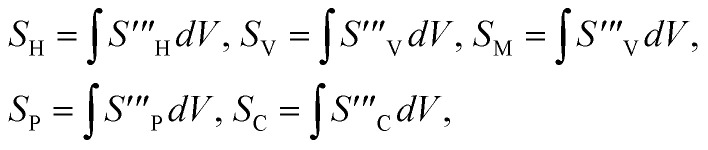


In the normalized notation, we obtain41
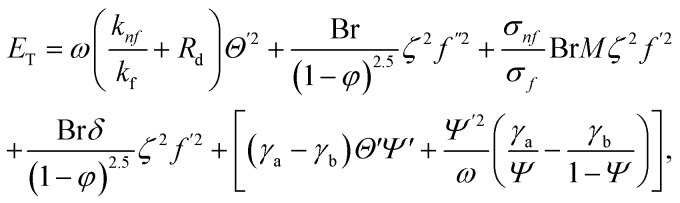


### 3.3. Dimensionless parameters

The following are dimensionless numbers, *i.e.*, porosity parameter, radiant parameter, magnetic parameter, Prandtl, Eckert, heat-generating parameter, Brinkman number, diffusion number for species and *X̂*, and *Ŷ* temperature ratio parameter.
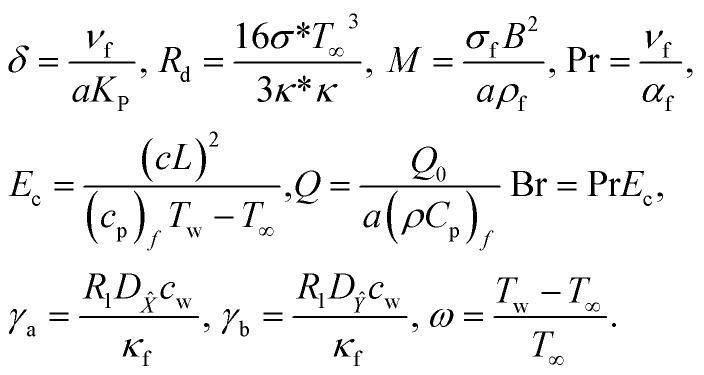


## 4. Numerical method and validation

For the dual diffusive flow specified by eqn (16), (17), and (23) under the wall and free stream boundary conditions (20), (21), and (24), the converted nonlinear ODE boundary value problem is a coupled multi-degree system of the seventh order. Because of the significant nonlinearity, a numerical solution is required. In order to solve this problem, MATLAB bvp5c solver was used. Providing adequate initial estimations for the meshes so that the solution converges to the target outcome is the most challenging aspect of solving a BVP. It directly controls the error in the calculation, whereas the more common bvp4c solver is unable to do so. This makes it a superior algorithm. This distinction between the solutions is less obvious at tighter error tolerances than that of Shampine and Kierzenka.^43^ The four-stage Lobatto IIIa collocation formula was implemented using “bvp5c”, a finite difference code, and the associated polynomial yields a *C*_1_ reliable solution that is uniformly precise for the 5th-order in [0, ∞]. The Runge–Kutta implicit formula was used to implement the procedure. The quantitative condensation found in the bvp4c was not used in the bvp5c. Bvp5c tickle with simple differential equations for the anonymous parameters as opposed to bvp4c, which directly deals with unknown parameters. The novel solver is based on the residual control for robustness. The residual was scaled to match the genuine error up to the fifth order of convergence. If the scaling residual was lower than a specified patience, the asymptotic error was likewise smaller than the tolerance, according to bvp5c,^43^ which was proven for a wide range of approaches. The value of *X*_MID_ at the midpoint and the slopes at both ends of the subinterval are interpolated with the bvp5c. The stepping formula described by Russel and Christansen^44^ was employed as follows:42
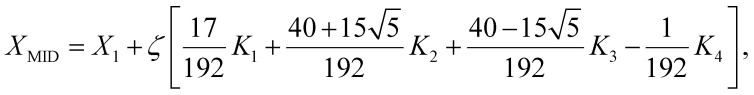
*X*_1_ is the initial guess in this case, and *K*_1_, *K*_2_, *K*_3_, and *K*_4_ are estimations with a stepping interval of *ζ* = 0.01. The algorithm has quick compilation times, is extremely effective, and delivers good accuracy. For each set of parameter values, the boundary layer region was reached while maintaining an iteration error of less than 10^−6^.

Furthermore, Tables 2 and 3 are created to compare the skin friction coefficient 
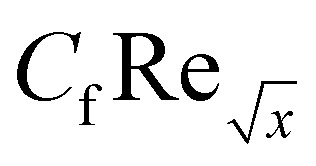
 and Nusselt number 
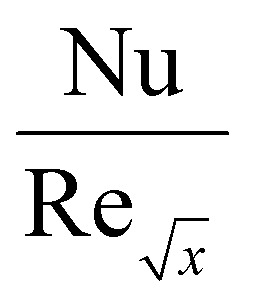
 in the absence of nanoparticles of Agand Al_2_O_3_, with the existing literature, in a limited sense, and was found to be in great agreement.

**Table 2:** Evaluation of skin friction coefficient values, when *φ* = 0, *δ* = 0

*M*	Ref. 45	Ref. 46	Present results
0	1.000	1.0000	1.0000
0.5	1.225	1.2247	1.2251
1.0	1.414	1.4142	1.4147
1.5	1.581	1.5811	1.5821
2.0	1.732	1.7321	1.7329

**Table 3:** Evaluation of Nusselt number values, when *C*_4_ = *R*_d_ = *E*_c_ = *Q* = 0

Pr	Ref. 47	Ref. 48	Ref. 46	Present results
0.07	0.0656	0.0656	0.0656	0.0657
0.20	0.1691	0.1691	0.1691	0.1693
0.70	0.4539	0.4539	0.4539	0.4541
2.00	0.9114	0.9114	0.9114	0.9117
7.00	1.8954	1.8954	1.8954	1.8957
20.0	3.3539	3.3539	3.3539	3.3539
70.0	6.4622	6.4622	6.4622	6.4626

The effects of various physical factors on the velocity profile, temperature and concentration profile, and entropy generation are discussed in detail in “Results and discussion”.

## 5. Results and discussion

**Fig. 2:**
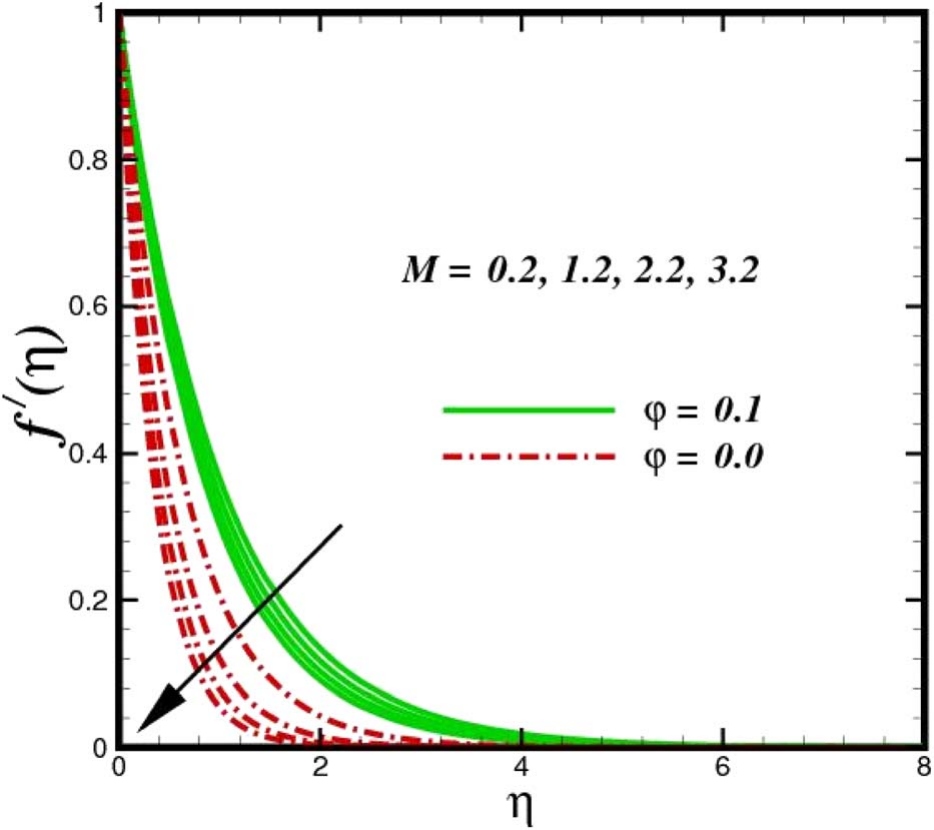
Velocity sketch *f*′(*η*) with the upshot in *M*.

**Fig. 3:**
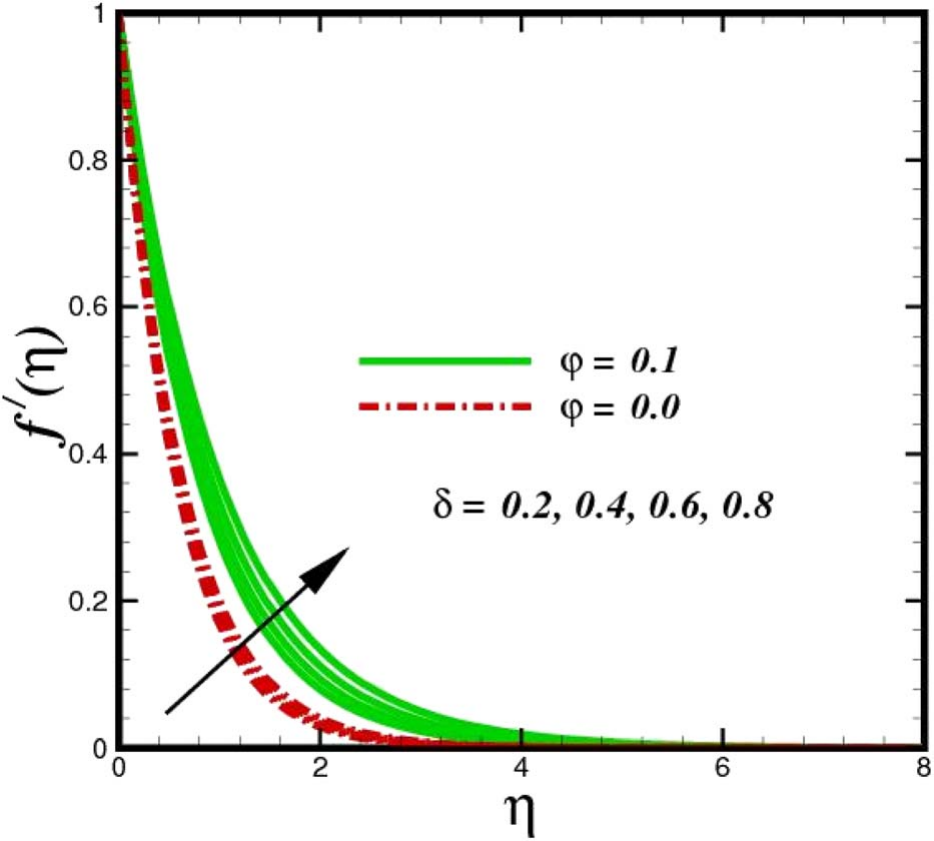
Velocity sketch *f*′(*η*) with the upshot in *δ*.

**Fig. 4:**
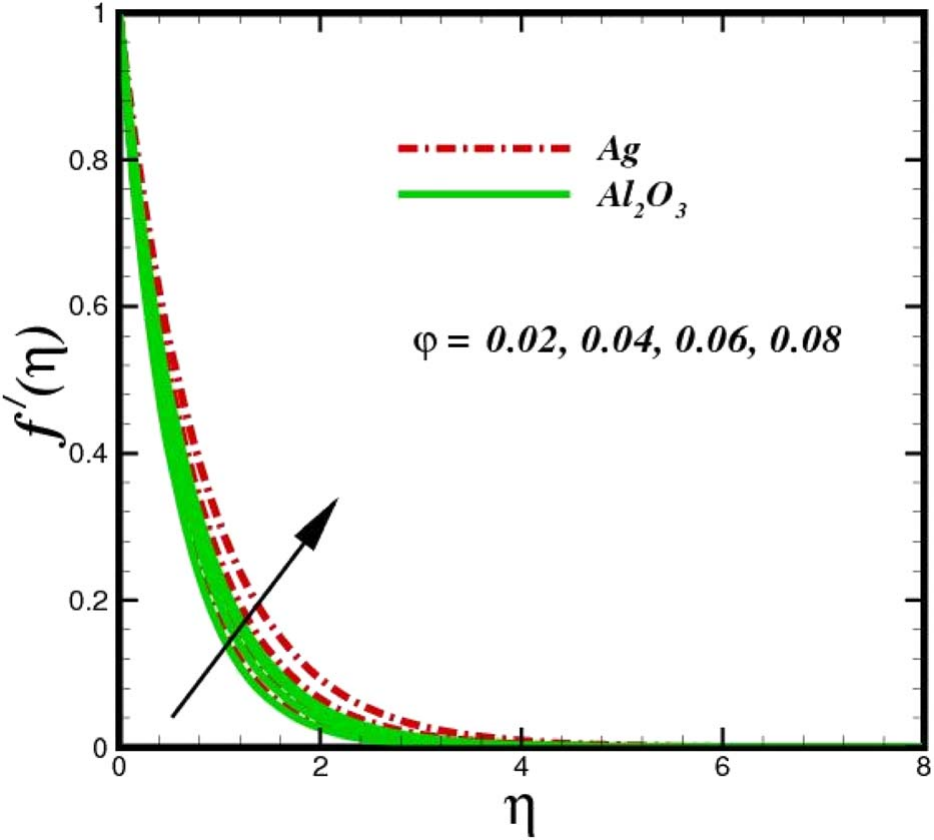
Velocity sketch *f*′(*η*) with the upshot in *φ*.

This section elaborates on the consequences of various emerging parameters, such as velocity, temperature, concentration, and entropy, providing physical justification. The structure of flow in the physical sense is illustrated through numerical simulations of velocities, temperatures, and concentration distributions against changes in physical limitations. The nanofluid velocity *f*′(*η*) profile is affected by the magnetic field parameter *M*, as shown in Fig. 2. The Lorentz force increases when an electric-conductive fluid is subjected to a magnetic field. The fluid rate decreases, since the fluid particles face an opposing force, and because of this opposed force, the fluid momentum slows down. Physically, the fluid particles travelling closer to the boundary layer under the influence of the Lorentz force slow down. The stretching sheet detracts the nanofluid velocity and also thickens the momentum boundary layer. This pattern results due to the stretching sheet, which made it more difficult for the molecules of the nanofluid to pass through. The fluid velocity can be controlled by a strong magnetic field, which is useful in applications, such as the production of hydromagnetic energy and the electromagnetic coating of wires. Fig. 3 illustrates how the velocity profile changes in response to the porosity parameter *δ*, keeping the fixed volumetric percentage of nanoparticles. A significant rise in velocity distribution is observed with augmented values of the porosity parameter *δ*. Physically, as the porosity parameter increases, the governing fluid experiences less friction, which boosts the fluid velocity. The effects of *φ*, or the solid volume fraction of nanoparticles, on the velocity profile *f*′(*η*) for two different types of nanoparticles (Al_2_O_3_–Ag) are shown in Fig. 4. Increasing the volume percentage of nanoparticles causes a drastic increase in the fluid viscosity, which enables the fluid to move more quickly. According to Fig. 4, the volume percentage of nanoparticles increases as the thickness of the momentum boundary layer decreases. Fluid velocity and gradient velocity both increase as a result. The graphs demonstrate that the silver Ag nanoparticles have a better velocity profile other than Al_2_O_3_. We observed a noticeable increase in velocity distribution as the volume percentage of nanoparticles increased.

**Fig. 5:**
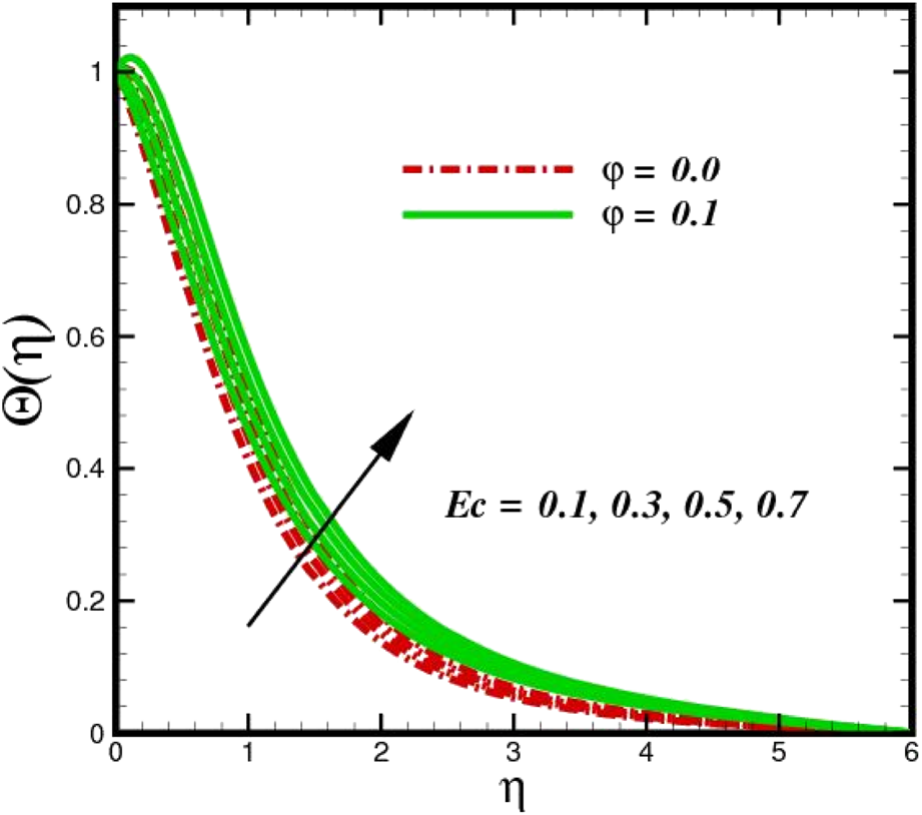
Temperature sketch *Θ*(*η*) with the upshot in *E*_c_.

**Fig. 6:**
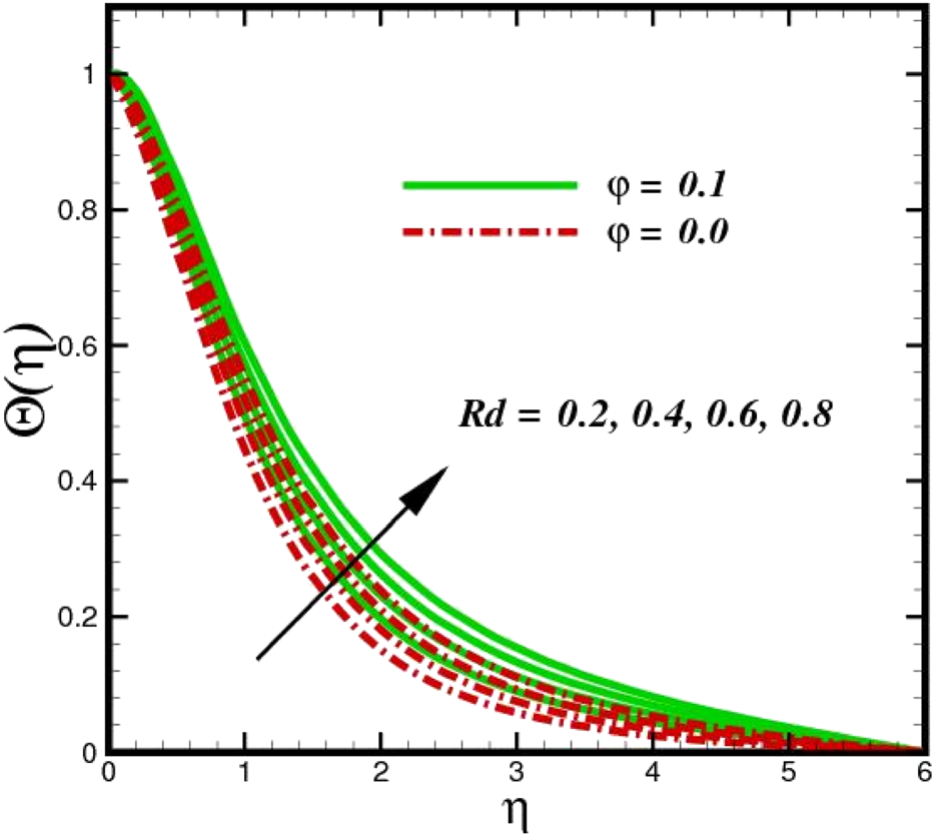
Temperature sketch *Θ*(*η*) with the upshot in *R*_d_.

**Fig. 7:**
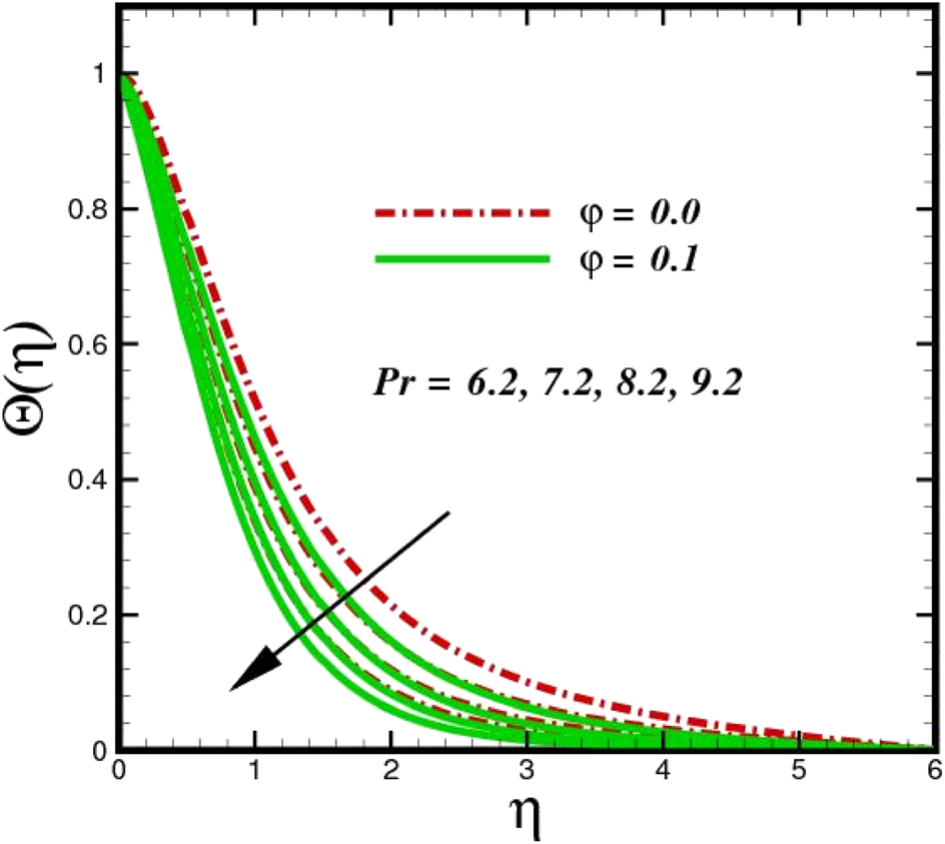
Temperature sketch *Θ*(*η*) with the upshot in Pr.

**Fig. 8:**
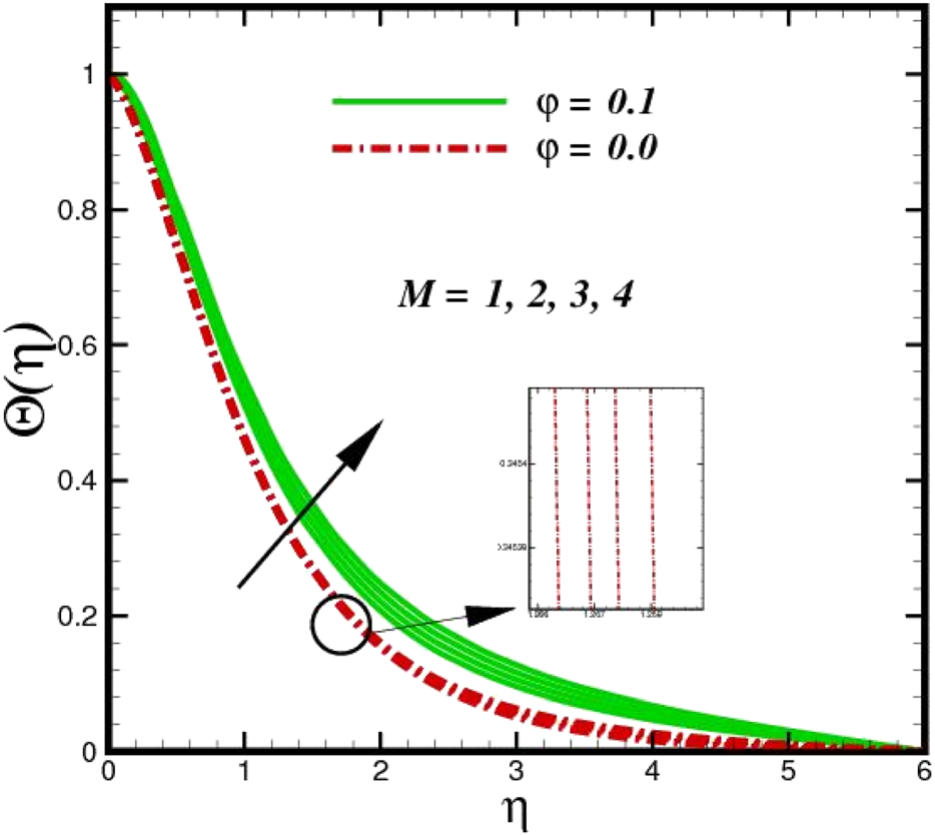
Temperature sketch *Θ*(*η*) with the upshot in *M*.

**Fig. 9:**
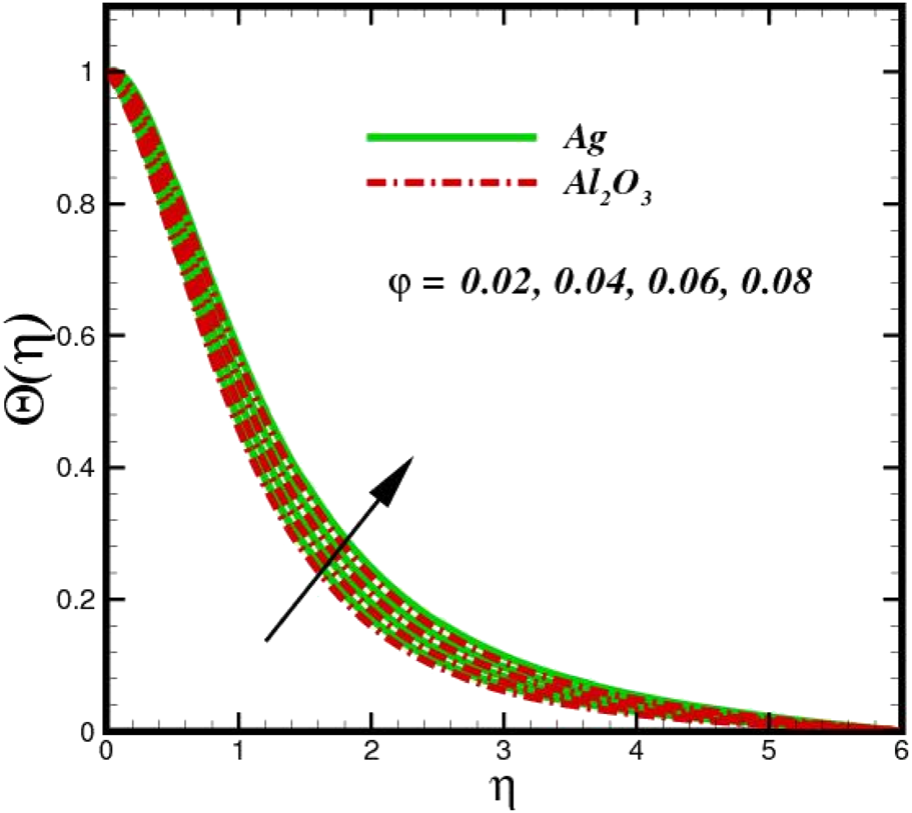
Temperature sketch *Θ*(*η*) with the upshot in *φ*.

Fig. 5 illustrates how the viscosity dissipation parameter *E*_c_ and two distinct nanoparticle morphologies (Al_2_O_3_–Ag) affect the temperature profile *Θ*(*η*). The friction between the fluid layers causes the temperature of nanofluids to rise as the *E*_c_ number rises. Physically, with viscous heating, the kinetic energy is converted into internal energy as a result of the increase in the temperature of nanofluid. Compared to Ag, the nanoparticles have a better temperature profile. The temperature profile of the aggregated nanoparticles is superior as compared to that of the ordinary fluid. Higher levels of friction result in the conversion of mechanical energy into thermal energy inside the fluid. From Fig. 6, it is evident that the radiation parameter R_d_ significantly increases the thermal boundary layer and the temperature profile. Physically, a higher radiation parameter results in additional heating as a result more heat is transferred to the corresponding fluid, increasing the thickness of the boundary layer and the temperature profile. The Rosseland radiative heat flux model is straightforward and limited to optically dense media capturing the effects of radiant heat transfer in systems for processing materials. The existing simulations offer a solid foundation for the development of more complex radiative flux models using the current model. Fig. 7 displays how the temperature profile varies for different Prandtl numbers. The thermal boundary layer and the temperature profile tend to be diminished by large values of the Prandtl number Pr. Physically, the temperature of fluids with lower thermal diffusivity is lower, as a result, the fluid exhibits a reduction in the temperature profile and boundary layer thickness. The energy transferred by molecular conduction is inhibited, which results in a reduction in the thermal profile and a reduction in the thickness of the thermal boundary layer as Pr is increased. The stretching sheet regime cooling is accomplished with greater Prandtl numbers. From a physical perspective, the Prandtl number links the heat diffusion rate and the momentum diffusion rate. As the Prandtl number rises (beyond unity), thermal diffusion is suppressed because the momentum diffusion rate is far greater than the thermal diffusion. The effect of the magnetic factor *M* on *Θ*(*η*)for the nanofluid is shown in Fig. 8. As the magnetic field intensity increases, the temperature profile increases. The thermal boundary layer thickness increases progressively as well. Physically, as the rate of the Lorentz force increases, the resistance to the motion of the nanoparticles increases, producing additional heat in the flow of the nanofluid. Fig. 9 depicts the relationship between the nanofluid thermal conductivity and temperature variation with regard to various values of volumetric fractions. The results show that as the thermal conductivity increases with increasing nanoparticle volumetric friction *φ*, the temperature increases. One of the elements influencing the enhancement of the thermal field of nanofluids is the intrinsic thermal conductivity of the nanofluid. Actually, by increasing the volumetric friction, the fluid molecular movement and the movement of the base fluid and nanoparticles are both accelerated. Energy is transferred from one layer of the nanofluid to another, increasing the thermal distribution of nanofluids.

**Fig. 10:**
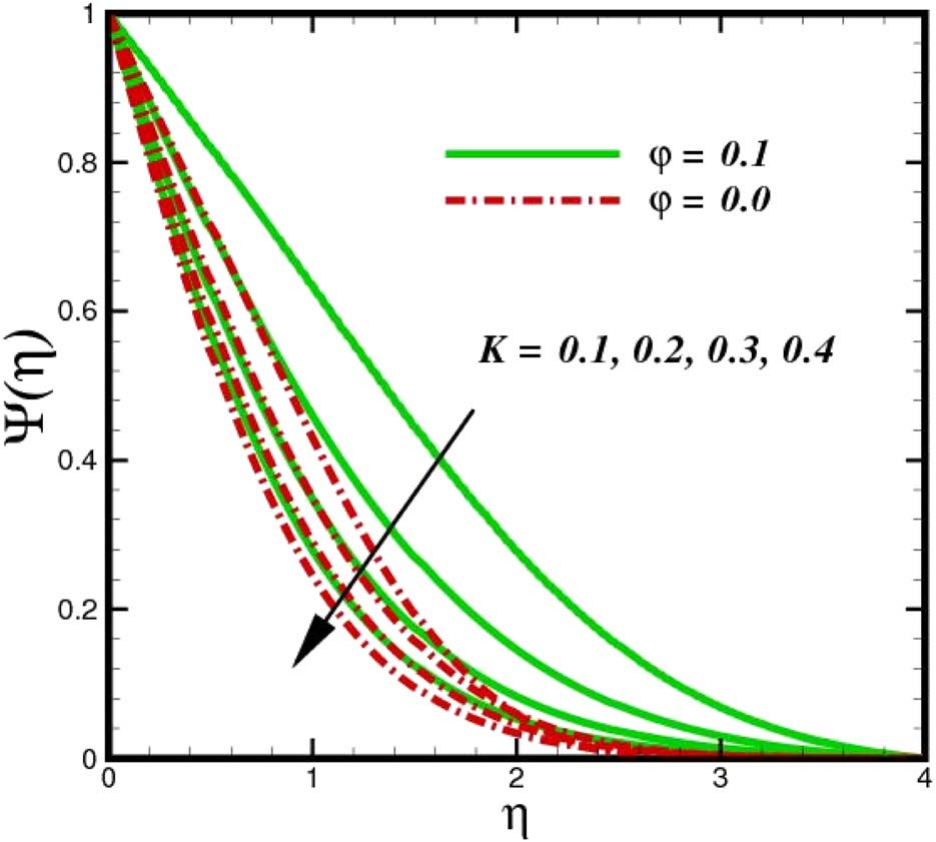
Species concentration *Ψ*(*η*) sketch with the upshot in *K*.

**Fig. 11:**
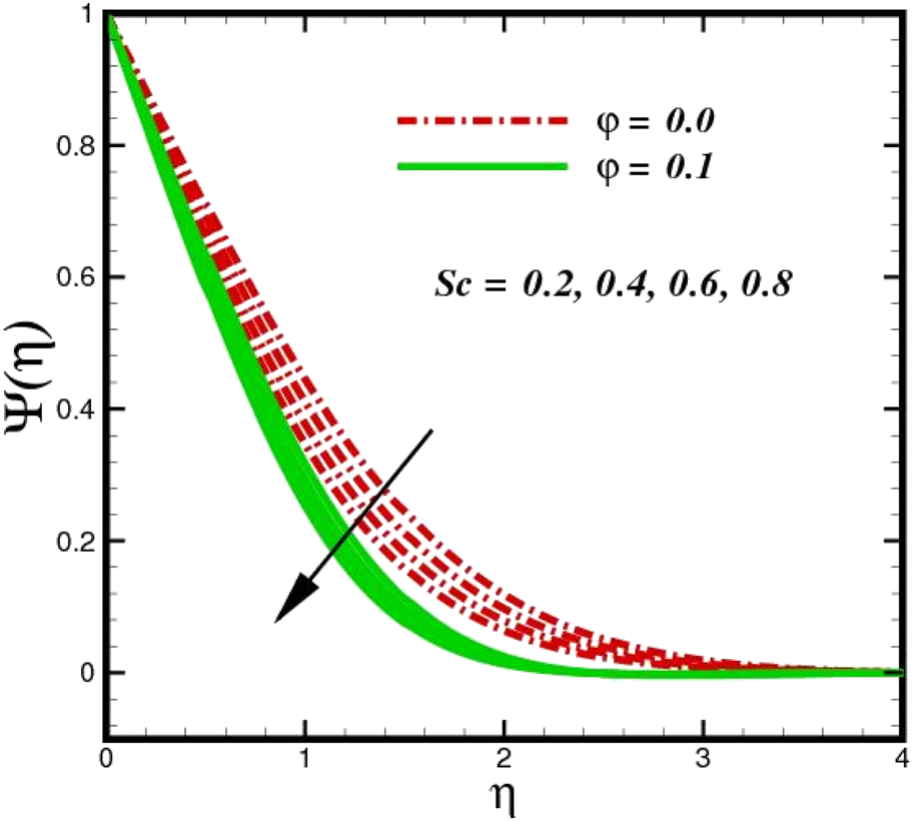
Species concentration *Ψ*(*η*) sketch with the upshot in Sc.

**Fig. 12:**
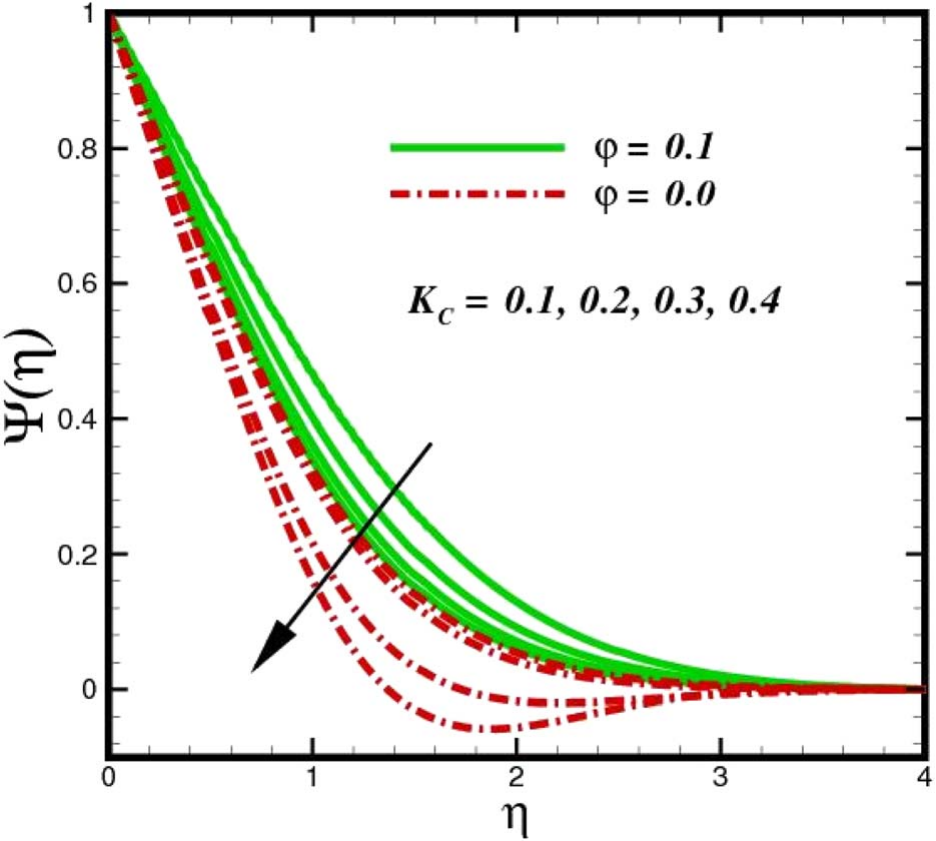
Species concentration *Ψ*(*η*) sketch with the upshot in *K*_C_.

As shown in Fig. 10, an increase in the homogeneous reaction parameter *K* is accompanied by a significant decrease in the concentration of species *X̂*. In the chemical reaction *X̂* → *Ŷ* the coupling term, −Sc*KΨ*(1 − *Ψ*)^2^ in eqn (20), the parameter *K*, appears. The species *Ŷ* boundary layer equation contains a related cross-link term, as shown in eqn (12). However, these concept polarities are diametrically opposite. The reaction is destructive (negative) when species *X̂* is used, whereas it is constructive when species *Ŷ* (positive) is used. As species *X̂* concentration magnitudes increase with increasing the distance from the stretched surface, a rapid ascent in species *X̂* concentration is observed with increasing transverse coordinates, as previously discussed. The impact of Sc on *Ψ*(*η*) is observed from the data in Fig. 11. The growth in Sc dwindles *Ψ*(*η*) in both scenarios. The smallest Sc < 0.1 represents the highest nanoparticle concentration. As Sc > 0.1 escalations begin, a reduction in concentration happens. Moreover, the NP aggregated fluid exhibits better mass transfer than ordinary fluid flows. Herein, we find that fluid flow without NP aggregation results in lower mass transfer. It is clear that rising Schmidt numbers significantly increase the concentration magnitudes, and this trend is consistent across the boundary layer of the stretched surface. In polymers, gaseous diffusion occurs when Sc > 1, therefore, the species diffusion rate exceeds the diffusion rate of momentum, leading to greater concentration magnitudes. In contrast, when Sc < 1, the species diffusion rate is exceeded by the momentum diffusion rate, reducing the concentration values. As the heterogeneous reaction parameter (*K*_C_) is increased, there is a noticeable suppression in the concentration magnitudes, as shown in Fig. 12. It appears in the conditions along the wall edge *Ψ*′(*ζ*,0) = *K*_C_*Ψ*(*ζ*,0). According to the autocatalytic reaction eqn (2), which states that *X̂* → *Ŷ*, rate *K*_s_*c*, it is connected to the first order, isothermal catalytic reaction at the substrate (stretched) surface. Physically, the highest conversion of species *X̂* to a new species occurs near the stretched surface resulting in minimizing the concentrations. The concentration of species *X̂* increases from the wall to the free stream, where it naturally reaches its maximum far away from the stretched surface. Since more and more species *X̂* are destroyed as *K*_s_ increases, species *X̂* concentration magnitudes (molecular diffusion) are inhibited. All profiles asymptotically converge to the free stream, supporting the simulations of an infinite boundary condition.

**Fig. 13:**
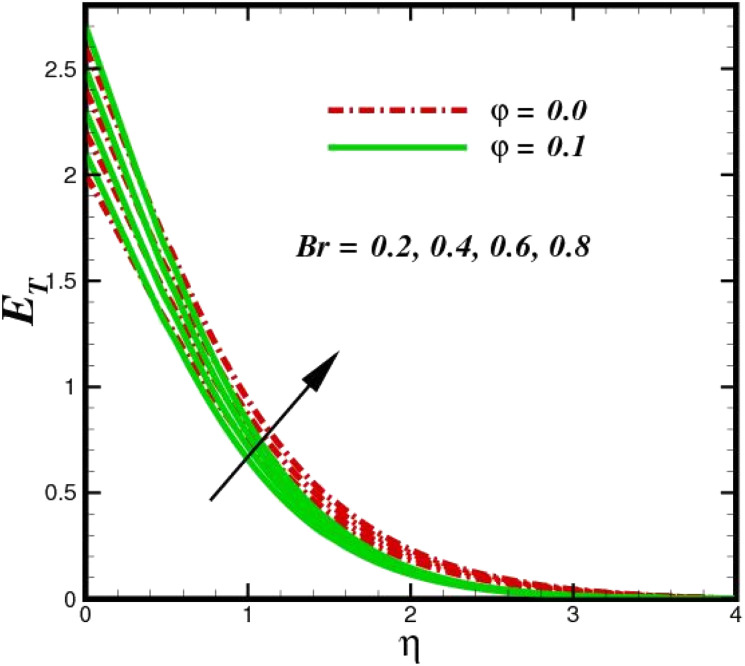
Total entropy *E*_T_ sketch with the upshot in Br.

**Fig. 14:**
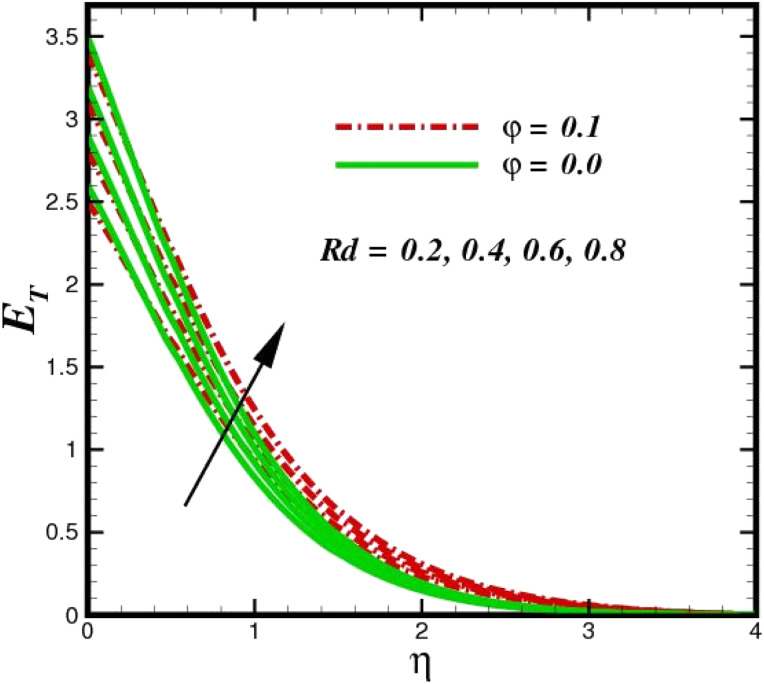
Total entropy *E*_T_ sketch with the upshot in *R*_d_.

**Fig. 15:**
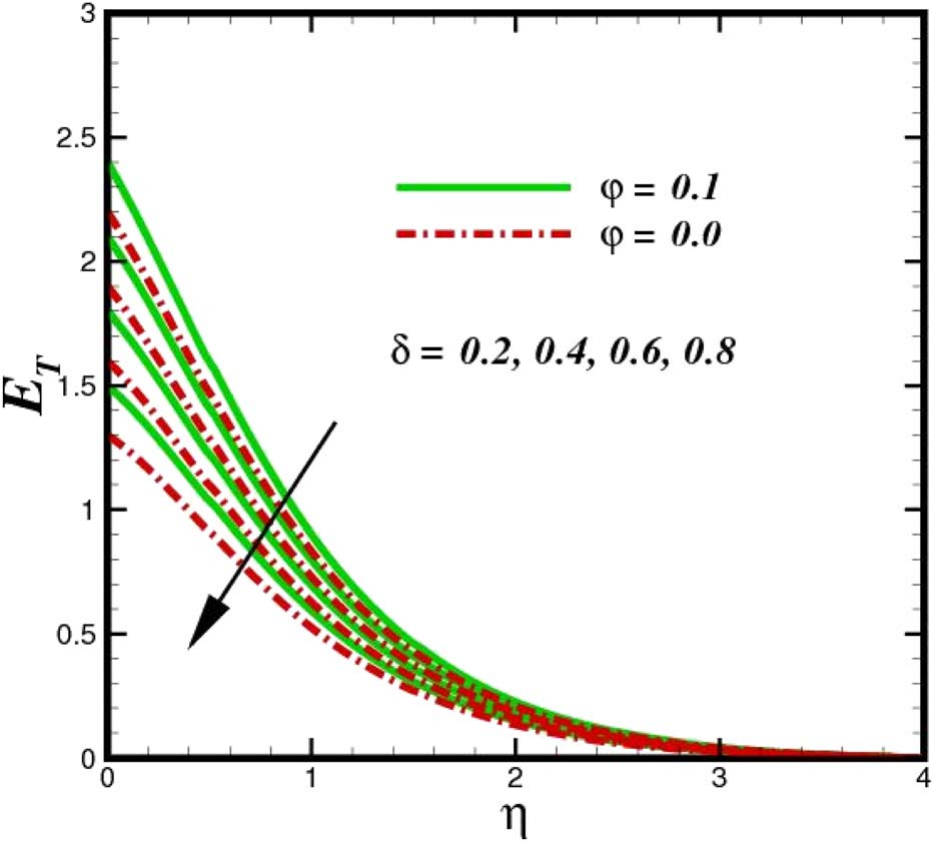
Total entropy *E*_T_ sketch with the upshot in *δ*.

**Fig. 16:**
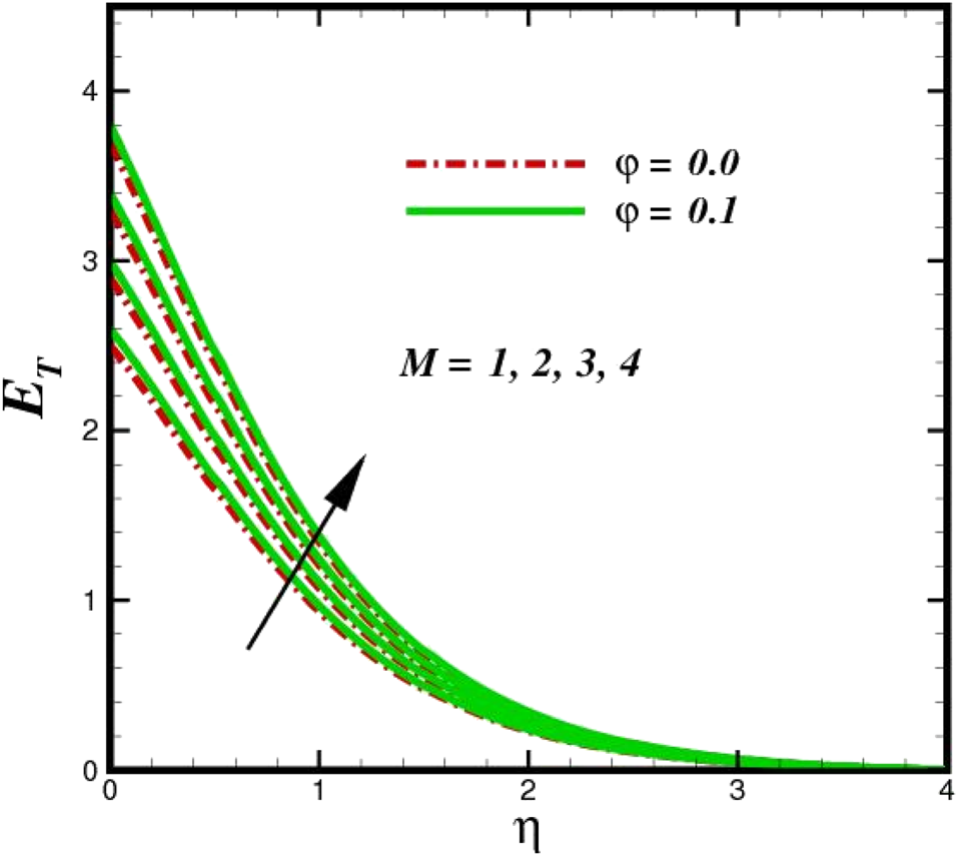
Total entropy *E*_T_ sketch with the upshot in *M*.

**Fig. 17:**
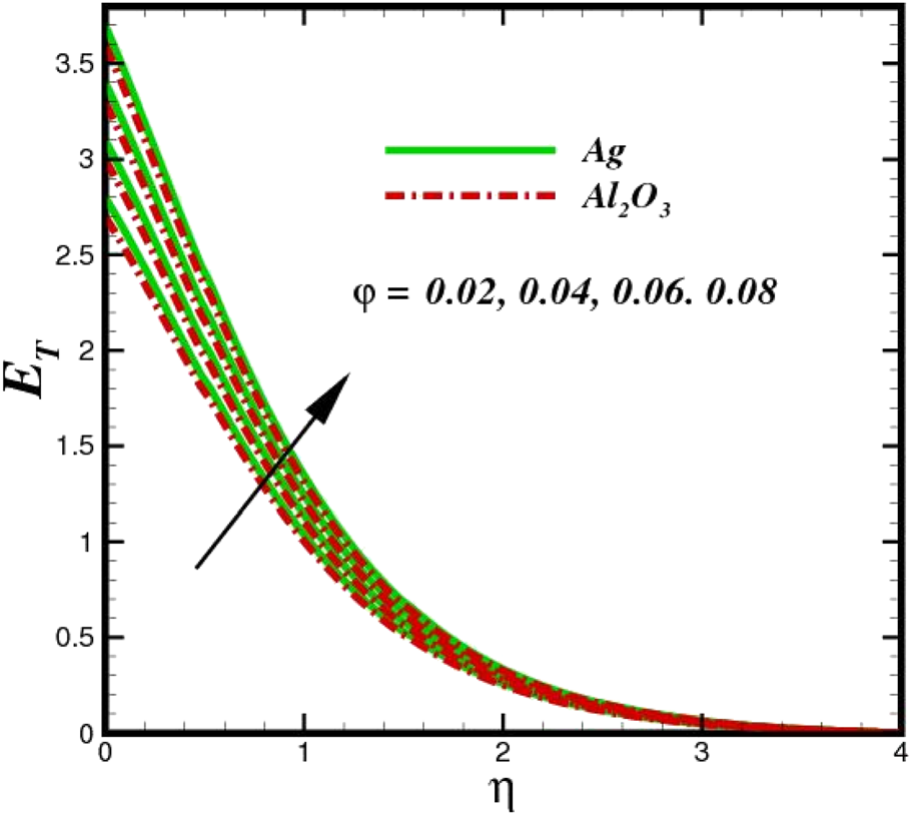
Total entropy *E*_T_ sketch with the upshot in *φ*.

**Fig. 18:**
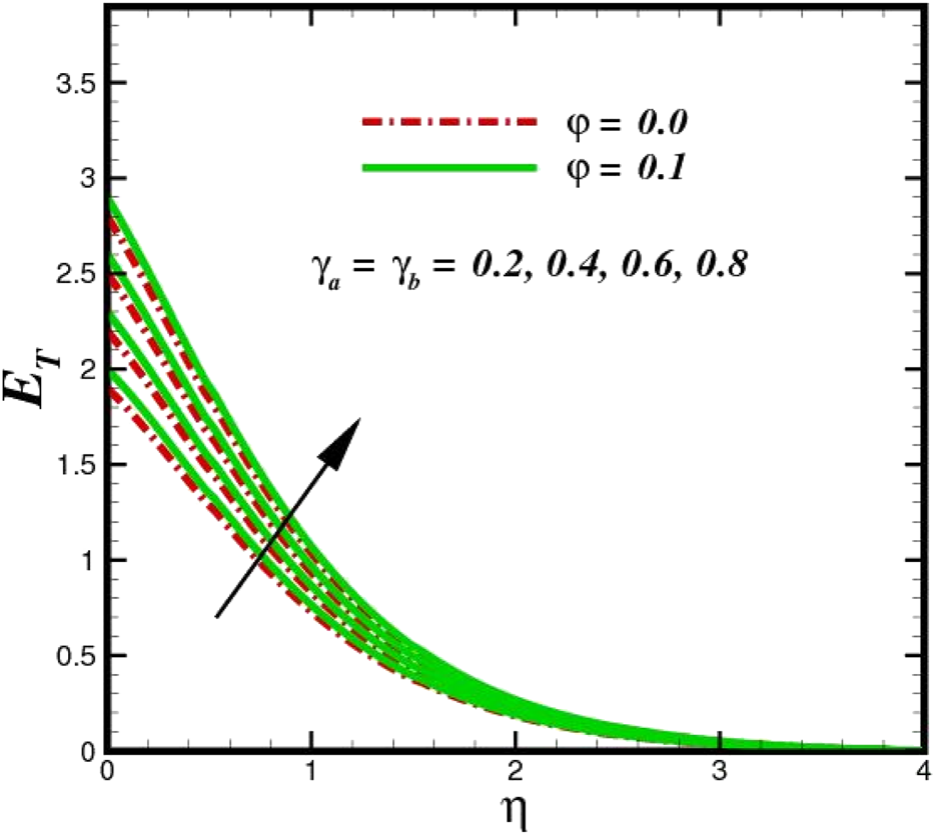
Total entropy *E*_T_ sketch with the upshot in *γ*_a_ = *γ*_a_.

The prominence of thermodynamics inquiry is to assess the temperature and heat variation losses, which detract the available energy. The concept of entropy is explained by the second law of thermodynamics. Concisely, entropy generation explains the dissipated energy within the thermal system, which consequently increases the level of disorder in the system. Entropy profiles *E*_T_ for various values of Brinkman number Br are shown in Fig. 13. It should be noted that the Brinkman number determines the relative significance of the viscous effect. It can be observed that the *E*_T_ increases with increasing Br values. A higher entropy is generated by fluid friction due to higher levels of Br. Furthermore, the impacts of entropy formation are stronger close to the sheet surface. When the fluid moves far away from the sheet surface, these effects become less noticeable. Since the Brinkman number is inversely proportional to the square of the sheet stretching velocity, the dimensionless entropy generation number is greater for the bigger values of Br on the sheet surface (Br ≥ 0.4). The stretching velocity of the sheet affects the fluid in such a way that at the surface of the sheet, the fluid accelerates and as a result entropy generation rate rises. For Br < 0.4, it is evident that *E*_T_ drops with increasing distance from the sheet. The influence of radiation on the entropy rate is shown in Fig. 14. Physically, a higher (*R*_d_) enhances the radiative emission, which intensifies the collision of fluid particles. From Fig. 15, it can be seen that the system rate of producing entropy decreases as the porosity parameter *δ* increases. As the non-linear drag increases, the system's internal entropy intensifies because of frictional heating. There is a considerable impact of fluid friction and heat transfer within the entropy production system for different values of *δ*. A lower temperature differential between the fluid and the stretched surface is obtained because of the concentrated frictional heating intensifications that drastically lower the fluid temperature. The increased temperature difference is achieved by *δ*, which increases the surface thermal transport rate from the fluid to the sheet walls. The entropy rate for magnetic parameters is illustrated in Fig. 16. Lorentz force increases when magnetic field intensity increases. Over the boundary layer region, significant heat generation occurs, and the system's overall disorderliness increases because of the strength of the magnetic field. For various fixed parameters, Fig. 17 shows the variation of frictional entropy generation in the laminar flow regime inside the heat sink with regard to the particle volumetric fraction. EG grows when the particle volume fraction is increased. However, there is a definite difference in the order of this increase. For instance, Al_2_O_3_ particles respond sharply to heat transport, which means that the EG produced by changes in the volume percentage of these particles rises because of large friction. Fig. 18 shows the corresponding entropy-generating field for various diffusivity variable inputs *γ*_a_ and *γ*_a_. This figure concludes that increasing the diffusivity coefficient inputs leads to an improvement in the entropy creation. The diffusion of the fluid particles increases with increasing levels of the diffusivity coefficient. In addition, there is a sharp increase in the profile as the value is increased.

## 6. Conclusion

In this study, the irreversibility analysis for the flow of a water-based nanofluid over a stretched surface was examined while considering the dual chemical species features and other physical factors. A complicated geometric stretched surface with many real-world engineering applications was used to model the flow. By dispersing the nanomaterial on the slit surface, the boundary layer separation from the surface was managed. The flow was subjected to external radiation, a heat source, and magnetic effects. In addition, the volumetric friction strategy described in the literature as a modified Tiwari-Das model (TDM) was used to execute this project. Applying computational fluid dynamics (CFD), a thorough examination and comparison of the thermo-hydraulic and entropy performance of a water-based nanofluid evacuated across a stretched surface was performed. H_2_O/Ag–Al_2_O_3_ nanofluid was used in the analysis. The numerical model accounts for the changes in operational parameters, including thermal radiation and mass flow rates. The model highlights the rate of entropy development due to the global and local forms of heat transmission, heat loss, and viscous effects. Using sets of sophisticated numerical techniques, including Bvp5c based on finite difference techniques, the governing PDEs (non-linear and coupled) were solved. The following concluding observations are made from the preceding thorough analysis:

1. An increment in velocity is noticed for both nanoparticles against the porosity factor, while the opposite consequence holds against the magnetic parameter.

2. Both, alumina (Al_2_O_3_) and silver (Ag) nanoparticles have nearly similar effects on thermal and fluid flow with an augmented volume fraction.

3. The Eckert number and radiation for both silver (Ag) and alumina (Al_2_O_3_) nanoparticles show an increase in temperature.

4. The aqueous concentration profile declines as the chemical reaction parameter and Schmidt increase.

5. Entropy generation grows as the Brinkman number, volumetric friction, diffusion parameter, and magnetic parameter, whereas it decreases with increasing porosity parameter.

6. The concentration of species decreases with homogenous and heterogeneous reaction rates.

7. By lowering the porosity within the sheet surface, the formation of entropy can be minimized.

8. The temperature gradient increases as the porosity parameter increases, but the velocity gradient shows the opposite pattern. The concentration gradient is reduced by increasing the values of the Schmidt number and the destructive reaction rate parameter, whereas a positive trend for the constructive parameter is revealed.

## Abbreviations

*a* > 0: Constant (s^−1^)
*K*_1_, *K*_s_: Homogeneous, heterogeneous reaction rate constants
*x*, *y*: Cartesian coordinates
*d*, *c*: Concentrations of the chemical species (m^2^ s^−1^)
*V⃑* = (*u*,*v*,0): Velocity field
*D*_T_, *D*_*X̂*_, *D*_*Ŷ*_: Diffusion coefficients (m^2^ s^−1^)
*V*_w_
: Wall velocity (m s^−1^)
(*ρc*_p_)_*f*_: Fluid heat capacitance
*u*_∞_
: Far-field velocity(m s^−1^)
(*ρc*_p_)_*p*_: Nanoparticle heat capacitance
*T*, *T*_w_, *T*_∞_: Fluid, wall and ambient temperature (K)
*R*_1_
: Molar gas constant (J K^−1^ mol^−1^
*X̂*, *Ŷ*: Dual chemical species
*f*
: Dimensionless velocity
*K*_P_
: Porosity of the porous medium
*Θ*
: Dimensionless temperature
*c*_p_
: Specific heat (J kg^−1^ K^−1^)
*χ*
: Dimensionless concentration
*k*_f_
: Thermal conductivity (Wm^−1^ K^−1^)
*τ*_w_
: Stress tensor component at the wall
*μ*_f_
: Dynamic viscosity (kg m^−1^ s^−1^)
*q*_w_
: Wall heat flux (Wm^−2^)
*Q*
: Heat source
*q*_M(*X̂*)_, *q*_M(*Ŷ*)_: Wall mass flux (kg m^−2^ s^−1^)

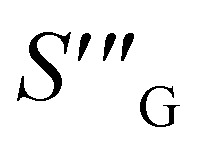

Entropy generation (Wm^−3^ K^−1^)
k*: Mean absorption coefficient (cm^−1^)
*σ*
: Electrical conductivity (S m^−1^)
*J*
: Current density (A m^−2^)
σ*: Stephan Boltzmann constant(Wm^−2^ K^−4^)
*B*_0_
: Magnetic field (Wm^2^)
*φ*_hf_ = *φ*_Ag_ + *φ*_Al_2_O_3__: Volume friction of *φ*_1_ and *φ*_2_
*ρ*_f_
: Fluid density (kg m^−3^)
*q*_r_
: Radiative heat flux
*ρ*_p_
: Nanoparticle's density (kg m^−3^)
*ζ*
: Similarity variable
*E*_T_
: Dimensionless entropy
Nu: Nusselt number
Br: Brinkman number
*Sh*_*X̂*_, *Sh*_*Ŷ*_: Sherwood numbers
*C*_f_
: Skin friction
Be: Bejan number

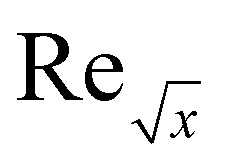

Reynold number
*γ*_b_
: Diffusion parameter
Pr: Prandtl number
*ω*
: Temperature ratio parameter
*E*_c_
: Eckert number
*M*
: Magnetic number
*R*_d_
: Radiation parameter
Sc: Schmidt number
*γ*_a_
: Diffusion parameter
*δ*
: Porosity parameter
*C*
: Fluid concentration (kg m^−3^)

## Data availability

The authors confirm that the data supporting the findings of this study are available within the manuscript.

## Author contributions

Conceptualization: Sohail Rehman, data curation, writing – review and editing, investigation: Hashim, solution methodology: Hashim, software: Serhan Alshammari, formal analysis: Ahmed Osman Ibrahim, Re-graphical representation and the addition of the analysis of data: Naeem Ullah, writing – original draft: Sohail Rehman, writing – review editing: Hashim, numerical process breakdown: Ahmed Osman Ibrahim, Re-modelling design: Serhan Alshammari.

## Conflicts of interest

The authors have no conflicts of interest.

## Supplementary Material
